# Hydrolyzed Collagen Induces an Anti-Inflammatory Response That Induces Proliferation of Skin Fibroblast and Keratinocytes

**DOI:** 10.3390/nu14234975

**Published:** 2022-11-23

**Authors:** Maysa Alves Rodrigues Brandao-Rangel, Carlos Rocha Oliveira, Fabiana Regina da Silva Olímpio, Flavio Aimbire, José Roberto Mateus-Silva, Felipe Augusto Chaluppe, Rodolfo P. Vieira

**Affiliations:** 1 Post-Graduate Program in Sciences of Human Movement and Rehabilitation, Federal University of Sao Paulo, Sao Jose dos Campos 12331-280, Brazil; 2 Post-Graduate Program in Biomedical Engineering, Federal University of Sao Paulo, Sao Jose dos Campos 12331-280, Brazil; 3 GAP Laboratory of Biotechnology, Sao Jose dos Campos 12243-020, Brazil; 4 Post-Graduate Program in Translational Medicine, Department of Medicine, Federal University of Sao Paulo, Sao Jose dos Campos 12331-280, Brazil; 5 PepTech Colágeno do Brasil Ltda, Development and Research Department, Jundiaí 13201-804, Brazil; 6 Post-Graduate Program in Bioengineering, Universidade Brasil, Sao Paulo 08230-030, Brazil; 7 Post-Graduate Program in Human Movement and Rehabilitation and in Pharmaceutical Sciences, Evangelical University of Goias (Unievangelica), Anapolis 75083-515, Brazil

**Keywords:** collagen, skin, fibroblast, keratinocytes, cytokines

## Abstract

Collagen-based products are found in different pharmaceuticals, medicine, food, and cosmetics products for a wide variety of applications. However, its use to prevent or improve the health of skin is growing dizzyingly. Therefore, this study investigated whether collagen peptides could induce fibroblast and keratinocyte proliferation and activation beyond reducing an inflammatory response induced by lipopolysaccharide (LPS). Human skin fibroblasts (CCD-1072Sk) and human keratinocytes (hKT-nh-skp-KT0026) were seeded at a concentration of 5 × 10^4^ cells/mL. LPS (10 ng/mL) and three doses of collagen peptides (2.5 mg/mL, 5 mg/mL, 10 mg/mL) were used. The readout parameters were cell proliferation; expression of inducible nitric oxide synthase (iNOS); expression of pro-collagen-1α by fibroblasts; and secretion of interleukin-1β (IL-1β), interleukin-6 (IL-6), interleukin-8 (IL-8), tumor necrosis factor α (TNF-α), transforming growth factor β (TGF-β), and vascular endothelial growth factor (VEGF) by both cell types. The results demonstrated that all doses of collagen supplementation induced increased proliferation of both human fibroblasts (*p* < 0.01) and human keratinocytes (*p* < 0.001), while only the dose of 10 mg/mL induced an increased expression of pro-collagen-1α by fibroblasts. Similarly, only the dose of 10 mg/mL reduced LPS-induced iNOS expression in fibroblasts (*p* < 0.05) and keratinocytes (*p* < 0.01). In addition, collagen supplementation reduced the LPS-induced IL-1β (*p* < 0.05), IL-6 (*p* < 0.001), IL-8 (*p* < 0.01), and TNF-α (*p* < 0.05), and increased the TGF-β and VEGF expression in fibroblasts. Furthermore, collagen supplementation reduced the LPS-induced IL-1β (*p* < 0.01), IL-6 (*p* < 0.01), IL-8 (*p* < 0.01), and TNF-α (*p* < 0.001), and increased the TGF-β (*p* < 0.05) and VEGF (*p* < 0.05) expression in keratinocytes. In conclusion, collagen peptides were found to induce fibroblast and keratinocyte proliferation and pro-collagen-1α expression, involving increased expression of TGF-β and VEGF, as well as the suppression of an inflammatory response induced by LPS.

## 1. Introduction

Skin is the largest organ in our bodies, and it is constituted by three primary layers: the epidermis, the dermis, and the hypodermis [1]. The skin’s main functions are to act as a barrier to prevent pathogens and other harmful agents from penetrating the body, as well as regulate body temperature and enable tactile sensations [1]. Beyond other cell types, skin fibroblasts are the main cell type present in skin connective tissue (dermis), presenting a crucial role as effector cells executing physiologic tissue repair, and pathological fibrogenesis leading to chronic fibrosing conditions in certain circumstances [2]. Skin fibroblasts also participate in the immune response of the skin, mainly releasing cytokines and growth factors [3]. During senescence, aging-induced immunosenescence predisposes inflammatory disturbances of the skin, including pruritic dermatoses and type 2 inflammation [3]. This immunosenescence is characterized by a chronic release of pro-inflammatory cytokines driving type 2 inflammatory dermatoses [3]. Therefore, scientists around the world are looking for strategies that are capable of preventing skin infections, especially in immunocompromised individuals [4]. In addition to skin fibroblasts, keratinocytes are cells with a vital function in the immune response of the skin, as well as their classical role in synthetizing keratin [5]. For instance, keratinocytes may be deeply hyperactivated by the bacteria *Staphylococcus aureus*, resulting in a huge synthesis and release of interleukin (IL) IL-6 and IL-8 [5], establishing an inflammatory response.

Furthermore, collagen is the main extracellular matrix protein in the skin structure [6]. Collagen synthesis changes during aging, with a reduction of 80 percent of type I collagen and 15 percent of type III collagen to a complete loss of type I collagen and type III collagen fibers becoming thicker and shorter [6]. Such alterations result in stiffening of the skin and a loss of humidity and elasticity, along with becoming more susceptible to infections [3,4,5,6].

Collagen-based products are found in a lot of pharmaceuticals, medicine, food, and cosmetics products for a wide variety of applications [7]. It may benefit several aspects of health, including wound healing, dental therapy, sarcopenia, bone defects, osteoarthritis, and rheumatoid arthritis, especially for aging people [7]. Therefore, knowledge and understanding of the effects of collagen-based products on different aspects of health and disease due to their increasing rate of use among athletes and individuals looking for aesthetic goals are urgently requested. However, so far, whether supplementation with collagen peptides may inhibit the inflammatory process in the skin through the activation of skin fibroblasts and keratinocytes is unknown. Therefore, the present study investigated whether collagen peptides may inhibit the inflammatory process induced by lipopolysaccharide (LPS) in skin fibroblasts and keratinocytes and whether collagen peptides may induce collagen synthesis by skin fibroblasts.

## 2. Material and Methods

### 2.1. Cell Lines and Experimental Design

Human fibroblasts (CCD-1072Sk) and human keratinocytes (hKT-nh-skp-KT0026) were purchased from the Cell Bank of Rio de Janeiro, Brazil. Both cell lineages were cultivated in a humid atmosphere in a 5% CO_2_ incubator at 37 °C. The cells were seeded at a concentration of 5 × 10^4^ cells/mL in a 24-well plate using the RPMI 1640 medium and 10% bovine fetal serum with a high amount of glucose. LPS from *Escherichia coli* (026:B6; L3755) was obtained from Sigma Aldrich, St. Louis, MO, USA). The experiments were done in triplicate and repeated once. Thus, the results represent the average of six individual wells ± the standard deviation.

### 2.2. Experimental Design

Human fibroblasts (CCD-1072Sk) and human keratinocytes (hKT-nh-skp-KT0026) were seeded at a concentration of 5 × 10^4^ cells/mL in a 24-well plate and the following conditions were created: (1) control (only medium stimulated), (2) lipopolysaccharide (LPS 10 ng/mL), (3) collagen 2.5 mg/mL, (4) collagen 5 mg/mL, (5) collagen 10 mg/mL, (6) LPS + collagen 2.5 mg/mL, (7) LPS + collagen 5 mg/mL, and (8) LPS + collagen 10 mg/mL. LPS was added for 1 h, followed by the addition of collagen in the doses described above for another 17 h to complete the total overnight period of 18 h. Hydrolyzed collagen (types I and III of hydrolyzed collagen) was kindly supplied by PeptPure^®^ (Peptpure, Jundiai, São Paulo, Brazil). Table 1 shows the physicochemical, microbiological, and molecular characteristics of the PeptPure^®^ collagen peptides.

### 2.3. Cell Proliferation Measurements

Briefly, the initial number of cells was previously known and seeded into the cell culture plates. After stimulations according to the experimental setup described above, the cells were carefully harvested and counted using the automated cell counter Countess 3 (Thermo Fisher Scientific, Waltham, MA, USA). The results were expressed for fibroblasts as the number of cells ×10^4^/mL and for keratinocytes as the number of cells ×10^5^/mL.

### 2.4. Inflammatory Mediators, Inducible Nitric Oxide Synthase (iNOS), and Pro-Collagen-1α Expression

Reverse transcription–quantitative PCR (RT-qPCR) was performed. Total RNA extracted from cell samples was converted to cDNA using a SuperScript^®^ III RT kit (Invitrogen, Carlsbad, CA, USA) according to the manufacturer’s protocol. The concentration of RNA was detected using a NanoDrop 2000 (Thermo Fisher Scientific, Waltham, MA, USA). GAPDH and 18S rRNA were used as the internal control. The thermocycling conditions were as follows: 95 °C for 10 min followed by 35 cycles of 95 °C for 15 s and 55 °C for 40 s. The 2^−ΔΔCq^ method was used to quantify the relative gene expression levels of the target genes. Relative standard curves were generated using serial dilutions, and all samples were run in triplicate [8]. The following sequences were used:

Pro-collagen-1α forward 5′-CGATGGATTCCAGTTCGAGTA-3′, reverse 5′-GTTTACAGGAAGCAGACAGG-3′ [9]; iNOS forward 5′-CTATCAGGAAGAAATGCAGGAGAT-3′, reverse 5′-GAGCACGCTGAGTACCTCATT-3′ [8]; IL-1β [5] forward 5′-GCAACTGTTCCTGAACTCAACT-3′, reverse 5′-ATCTTTTGGGGTCCGTCAACT-3′; IL-6 [4] forward 5′-AACCTGAACCTTCCAAAGATGG-3′, reverse 5′-TCTGGCTTGTTCCTCACTACT-3′; IL-8 [4] forward 5′-CATACTCCAAACCTTTCCACCCC-3′, reverse 5′-TCAGCCCTCTTCAAAAACTTCTCCA-3′; tumor necrosis factor α (TNF-α) [5] forward 5′-CTGAACTTCGGGGTGATCGG-3′, reverse 5′-GGCTTGTCACTCGAATTTTGAGA-3′; and vascular endothelial growth factor (VEGF) [3] forward 5′-TGCAGATTATGCGGATCAAACC-3′, reverse 5′-TGCATTCACATTTGTTGTGCTGTAG-3′.

### 2.5. Cell Proliferation

Cell (human fibroblasts (CCD-1072Sk) and human keratinocytes (hKT-nh-skp-KT0026)) proliferation was calculated by subtracting the number of cells obtained at the end of the experiment from the number of cells initially placed (5 × 10^4^ cells/mL). Thus, the results were expressed as the number of fibroblasts (CCD-1072Sk) and human keratinocytes (hKT-nh-skp-KT0026) per milliliter.

### 2.6. Cytokines and Growth Factors Measurement

The supernatant obtained from skin fibroblasts and keratinocytes was used to measure the levels of IL-1β (DY201), IL-6 (DY206), IL-8 (DY208), TNF-α (DY210), transforming growth factor β1 (TGF-β1) (DY240), and VEGF (DY293) using Duo Set ELISA kits from R&D Systems according to the manufacturer’s recommendations and using a microplate reader SpectraMax I3 (Molecular Devices, San Jose, CA, USA). The results were expressed in pg/mL.

### 2.7. Nitrite (NO_2_) and Nitrate (NO_3_) Measurements

NO_2_ and NO_3_ were measured in the supernatants of the cell culture of skin fibroblasts and keratinocytes using the Griess method through the Nitric Oxide Colorimetric Detection Kit cod r K023-H1 (Arbor Assays™, Ann Arbor, MI, USA) according to the manufacturer’s recommendations using a microplate reader SpectraMax I3 (Molecular Devices, San Jose, CA, USA). The results were expressed in μM/mL.

### 2.8. Statistical Analysis

GraphPad Prism 5.0 software (GraphPad Software, Inc., La Jolla, CA, USA) was used to perform the statistical analysis and to build the graphs. The distribution of the data was performed using Pearson’s test. The data presenting the parametric distribution were evaluated using one-way ANOVA followed by Newman–Keuls test for multiple comparisons between the groups. The data with a non-parametric distribution were evaluated using ANOVA on ranks followed by Dunn’s test for multiple comparisons between groups. A *p* < 0.05 was considered statistically significant.

## 3. Results

### 3.1. Effects of Collagen Supplementation on Cell Proliferation

**Figure 1:**
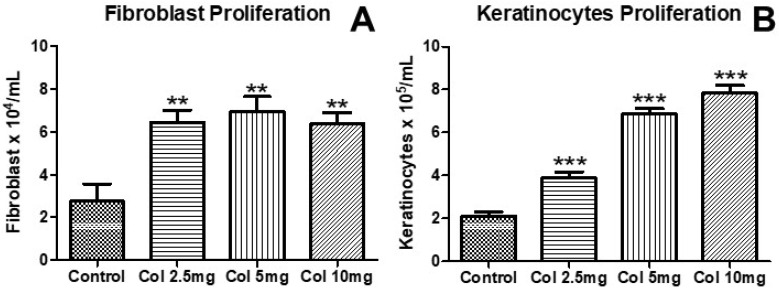
The effects of collagen supplementation on human fibroblast (**A**) and human keratinocyte (**B**) proliferation. For (**A**), ** *p* < 0.01 compared with the control group. For (**B**), *** *p* < 0.001 compared with the control group. Col, collagen.

Figure 1 shows the effects of collagen supplementation on human fibroblast (CCD-1072Sk) (Figure 1A) and human keratinocyte (hKT-nh-skp-KT0026) (Figure 1B) proliferation. The results demonstrated that all doses of collagen supplementation (2.5 mg/mL, 5 mg/mL, 10 mg/mL) increased the proliferation of both human fibroblasts (CCD-1072Sk) (Figure 1A, *p* < 0.01) and human keratinocytes (hKT-nh-skp-KT0026) (Figure 1A, *p* < 0.001).

### 3.2. Effects of Collagen Supplementation on the Expression of Pro-Collagen-1α and iNOS

**Figure 2:**
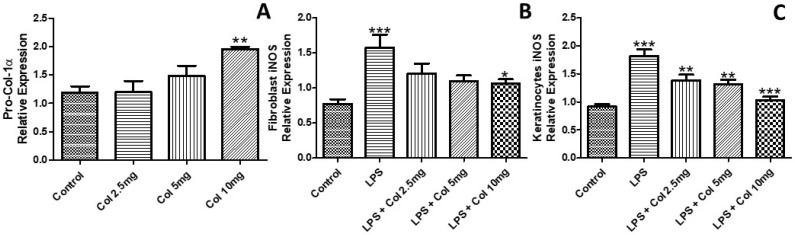
The effects of collagen supplementation on human fibroblasts synthesis of pro-collagen-1α (**A**) and on the expression of inducible nitric oxide synthase (iNOS) on fibroblasts (**B**) and keratinocytes (**C**). For (**A**), ** *p* < 0.01 compared with the control group. For (**B**), * *p* < 0.05 compared with the control and LPS groups; *** *p* < 0.001. For (**C**), *** *p* < 0.001 compared with the control and LPS groups and ** *p* < 0.01 compared with the LPS group. Pro-Col-1α, pro-collagen-1α; LPS, lipopolysaccharide.

Figure 2 shows the effects of collagen supplementation on human fibroblasts (CCD-1072Sk) activation through the expression of pro-collagen-1α (pro-col-1α) (Figure 2A) and iNOS (Figure 2B). The results demonstrated that only the dose of 10 mg/mL of collagen supplementation induced an increase in the expression of pro-col-1α in fibroblasts (Figure 2A, *p* < 0.01). On the other hand, only 10 mg/mL of collagen reduced LPS-induced iNOS expression in fibroblasts (Figure 2B, *p* < 0.05). Concerning the response of keratinocytes, the three studied doses (2.5 mg/mL, Figure 2C, *p* < 0.01), (5 mg/mL, Figure 2C, *p* < 0.01), and (10 mg/mL, Figure 2C, *p* < 0.001) of collagen reduced the LPS-induced iNOS expression.

### 3.3. Effects of Collagen Supplementation on Cytokine Gene Expression in Fibroblasts

**Figure 3:**
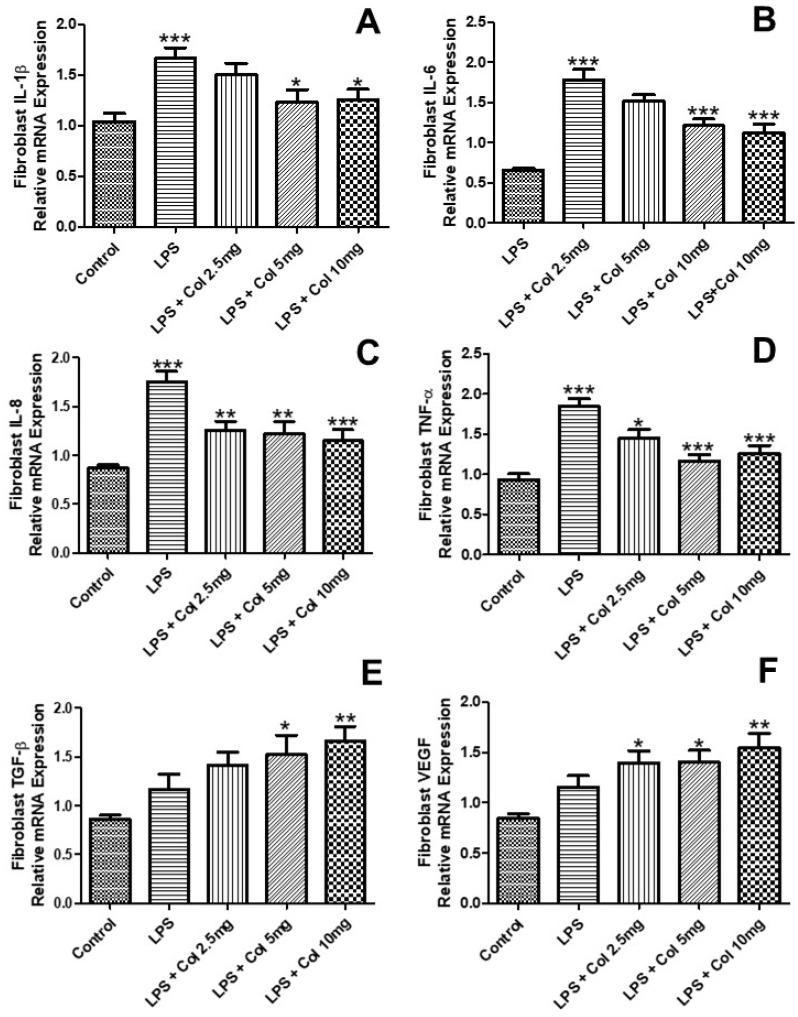
The effects of collagen supplementation on the mRNA expression of IL-1β (**A**), IL-6 (**B**), IL-8 (**C**), TNF-α (**D**), TGF-β (**E**), and VEGF (**F**) of human fibroblasts. For (**A**), *** *p* < 0.001 compared with the control group and * *p* < 0.05 compared with the LPS group. For (**B**), *** *p* < 0.001 compared with the control and LPS groups. For (**C**), *** *p* < 0.001 compared with the control and LPS groups and ** *p* < 0.01 compared with the LPS group. For (**D**), *** *p* < 0.001 compared with the control and LPS groups and * *p* < 0.05 compared with the LPS group. For (**E**,**F**), ** *p* < 0.01 and * *p* < 0.05 compared with the control group. IL-1β, interleukin-1β; IL-6, interleukin-6; IL-8, interleukin-8; TNF-α, tumor necrosis factor α; TGF-β, transforming growth factor β; VEGF, vascular endothelial growth factor.

Figure 3 shows the effects of collagen supplementation on the mRNA expression of IL-1β (Figure 3A), IL-6 (Figure 3B), IL-8 (Figure 3C), TNF-α (Figure 3D), TGF-β (Figure 3E), and VEGF (Figure 3F) of human fibroblasts (CCD-1072Sk). The results (Figure 3A) showed that LPS increased the expression of IL-1β (*p* < 0.001), while the doses of 5 mg/mL (*p* < 0.05) and 10 mg/mL (*p* < 0.05) of collagen significantly inhibited its expression. The results (Figure 3B) showed that LPS increased the expression of IL-6 (*p* < 0.001), while the doses of 5 mg/mL (*p* < 0.001) and 10 mg/mL (*p* < 0.001) of collagen significantly inhibited its expression. The results (Figure 3C) showed that LPS increased the expression of IL-8 (*p* < 0.001), while the doses of 2.5 mg/mL (*p* < 0.01), 5 mg/mL (*p* < 0.001), and 10 mg/mL (*p* < 0.001) of collagen significantly inhibited its expression. The results (Figure 3D) showed that LPS increased the expression of TNF-α (*p* < 0.001), while the doses of 2.5 mg/mL (*p* < 0.05), 5 mg/mL (*p* < 0.001), and 10 mg/mL (*p* < 0.001) of collagen significantly inhibited its expression. The results (Figure 3E) showed that LPS + collagen 5 mg/mL (*p* < 0.05) and LPS + collagen 10 mg/mL (*p* < 0.01) increased the expression of TGFβ. The results (Figure 3F) showed that LPS + collagen 2.5 mg/mL (*p* < 0.05), collagen 5 mg/mL (*p* < 0.05), and LPS + collagen 10 mg/mL (*p* < 0.01) increased the expression of VEGF.

### 3.4. Effects of Collagen Supplementation on Cytokine Gene Expression in Keratinocytes

**Figure 4:**
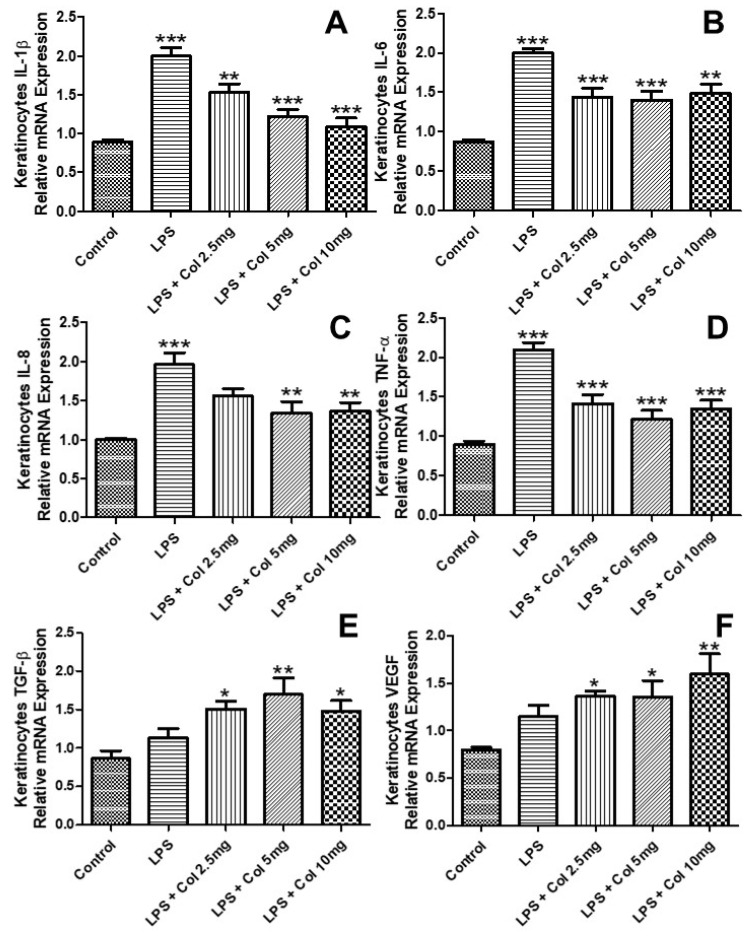
The effects of collagen supplementation on the mRNA expression of IL-1β (**A**), IL-6 (**B**), IL-8 (**C**), TNF-α (**D**), TGF-β (**E**), and VEGF (**F**) of human keratinocytes. For (**A**), *** *p* < 0.001 compared with the control group and LPS group and ** *p* < 0.01 compared with the LPS group. For (**B**), ** *p* < 0.01; *** *p* < 0.001 compared with the control and LPS groups. For (**C**), *** *p* < 0.001 compared with the control group and ** *p* < 0.01 compared with the LPS group. For (**D**), *** *p* < 0.001 compared with the control and LPS groups. For (**E**,**F**), ** *p* < 0.01 and * *p* < 0.05 compared with the control group.

Figure 4 shows the effects of collagen supplementation on the mRNA expression of IL-1β (Figure 4A), IL-6 (Figure 4B), IL-8 (Figure 4C), TNF-α (Figure 4D), TGF-β (Figure 4E), and VEGF (Figure 4F) of human fibroblasts (CCD-1072Sk). The results (Figure 4A) showed that LPS increased the expression of IL-1β (*p* < 0.001), while the doses of 5 mg/mL (*p* < 0.05) and 10 mg/mL (*p* < 0.05) of collagen significantly inhibited its expression. The results (Figure 4B) showed that LPS increased the expression of IL-6 (*p* < 0.001), while the doses of 5 mg/mL (*p* < 0.001) and 10 mg/mL (*p* < 0.001) of collagen significantly inhibited its expression. The results (Figure 4C) showed that LPS increased the expression of IL-8 (*p* < 0.001), while the doses of 2.5 mg/mL (*p* < 0.01), 5 mg/mL (*p* < 0.01), and 10 mg/mL (*p* < 0.001) of collagen significantly inhibited its expression. The results (Figure 4D) showed that LPS increased the expression of TNF-α (*p* < 0.001), while the doses of 2.5 mg/mL (*p* < 0.05), 5 mg/mL (*p* < 0.001), and 10 mg/mL (*p* < 0.001) of collagen significantly inhibited its expression. The results (Figure 4E) showed that LPS + collagen 5 mg/mL (*p* < 0.05) and LPS + collagen 10 mg/mL (*p* < 0.01) increased the expression of TGF-β. The results (Figure 4F) showed that LPS + collagen 2.5 mg/mL (*p* < 0.05), collagen 5 mg/mL (*p* < 0.05), and LPS + collagen 10 mg/mL (*p* < 0.01) increased the expression of VEGF.

### 3.5. Effects of Collagen Supplementation on Cytokine Protein Levels in Fibroblasts

**Figure 5:**
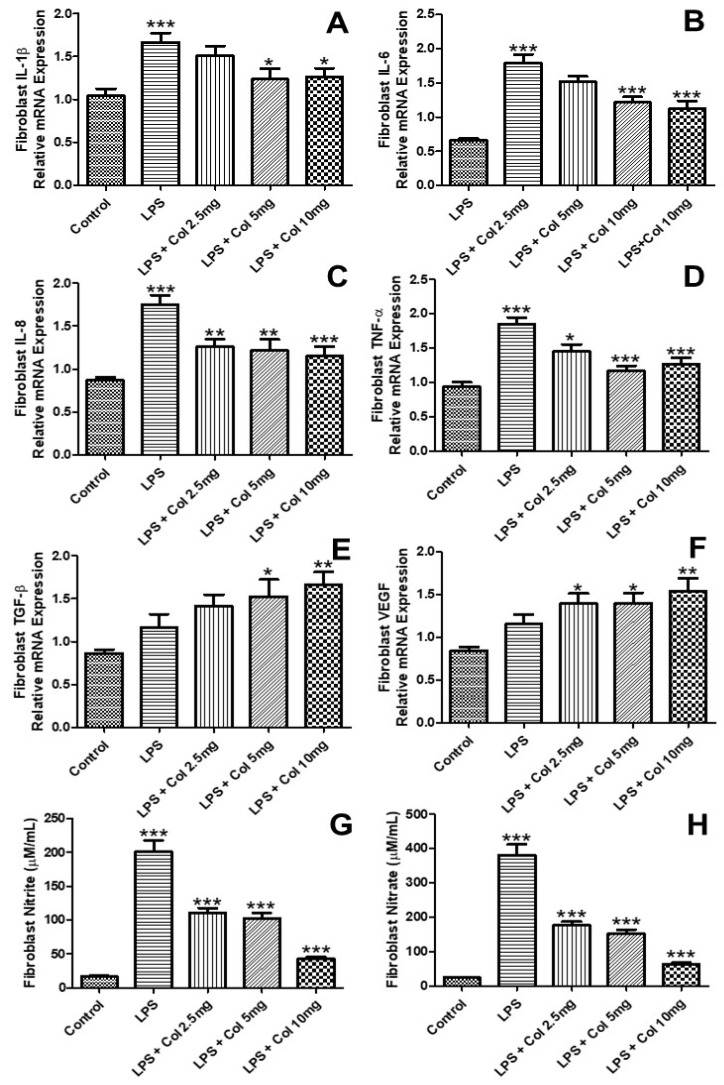
The effects of collagen supplementation on the levels of IL-1β (**A**), IL-6 (**B**), IL-8 (**C**), TNF-α (**D**), TGF-β (**E**), VEGF (**F**), nitrite (**G**), and nitrate (**H**) of human keratinocytes. For (**A**), *** *p* < 0.001 compared with the control group and LPS group and * *p* < 0.05 compared with the LPS group. For (**B**), *** *p* < 0.001 compared with the control and LPS groups. For (**C**), *** *p* < 0.001 compared with the control and LPS groups and ** *p* < 0.01 compared with the LPS group. For (**D**), *** *p* < 0.001 compared with the control and LPS group and * *p* < 0.05 compared with the LPS group. For (**E**,**F**), ** *p* < 0.01 compared with the LPS groups and * *p* < 0.05 compared with the LPS group. For (**G**,**H**), *** *p* < 0.001 compared with the control and LPS groups.

Figure 5 shows the effects of collagen supplementation on the levels of IL-1β (Figure 5A), IL-6 (Figure 5B), IL-8 (Figure 5C), TNF-α (Figure 5D), TGF-β (Figure 5E), and VEGF (Figure 5F) of human fibroblasts (CCD-1072Sk). The results (Figure 5A) showed that LPS increased the levels of IL-1β (*p* < 0.001), while the doses of 2.5 mg/mL (*p* < 0.05), 5 mg/mL (*p* < 0.001), and 10 mg/mL (*p* < 0.001) of collagen significantly inhibited its levels. The results (Figure 5B) showed that LPS increased the levels of IL-6 (*p* < 0.001), while the doses of 2.5 mg/mL (*p* < 0.01), 5 mg/mL (*p* < 0.001), and 10 mg/mL (*p* < 0.001) of collagen significantly inhibited its levels. The results (Figure 5C) showed that LPS increased the levels of IL-8 (*p* < 0.001), while the doses of 5 mg/mL (*p* < 0.001) and 10 mg/mL (*p* < 0.001) of collagen significantly inhibited its levels. The results (Figure 5D) showed that LPS increased the levels of TNF-α (*p* < 0.001), while the doses of 2.5 mg/mL (*p* < 0.01), 5 mg/mL (*p* < 0.001), and 10 mg/mL (*p* < 0.001) of collagen significantly inhibited its levels. The results (Figure 5E) showed that LPS (*p* < 0.001), LPS + collagen 2.5 mg/mL (*p* < 0.001), LPS + collagen 5 mg/mL (*p* < 0.001), and LPS + collagen 10 mg/mL (*p* < 0.001) increased the levels of TGF-β in comparison with the control group. The results (Figure 5F) showed that LPS (*p* < 0.001), LPS + collagen 2.5 mg/mL (*p* < 0.001), LPS + collagen 5 mg/mL (*p* < 0.001), and LPS + collagen 10 mg/mL (*p* < 0.001) increased the levels of VEGF in comparison with the control group.

### 3.6. Effects of Collagen Supplementation on Cytokine Protein Levels in Keratinocytes

**Figure 6:**
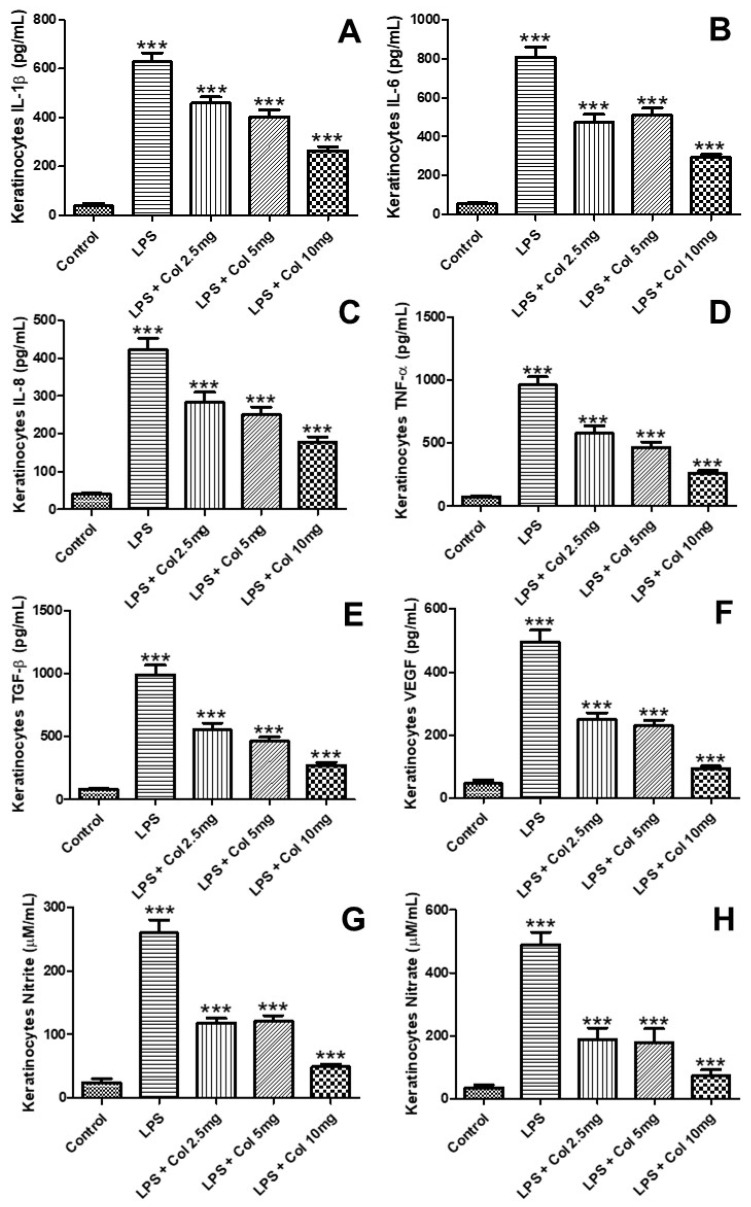
The figure shows the effects of collagen supplementation on the levels of IL-1β (**A**), IL-6 (**B**), IL-8 (**C**), TNF-α (**D**), TGF-β (**E**), VEGF (**F**), nitrite (**G**), and nitrate (**H**) of human keratinocytes. For (**A**–**H**), *** *p* < 0.001 compared with the control group and LPS group.

Figure 6 shows the effects of collagen supplementation on the levels of IL-1β (Figure 6A), IL-6 (Figure 6B), IL-8 (Figure 6C), TNF-α (Figure 6D), TGF-β (Figure 6E), and VEGF (Figure 6F) of human fibroblasts (CCD-1072Sk). The results (Figure 6A) showed that LPS increased the levels of IL-1β (*p* < 0.001), while the doses of 2.5 mg/mL (*p* < 0.001), 5 mg/mL (*p* < 0.001), and 10 mg/mL (*p* < 0.001) of collagen significantly inhibited its levels. The results (Figure 6B) showed that LPS increased the levels of IL-6 (*p* < 0.001), while the doses of 2.5 mg/mL (*p* < 0.01), 5 mg/mL (*p* < 0.001), and 10 mg/mL (*p* < 0.001) of collagen significantly inhibited its levels. The results (Figure 6C) showed that LPS increased the levels of IL-8 (*p* < 0.001), while the doses of 5 mg/mL (*p* < 0.001) and 10 mg/mL (*p* < 0.001) of collagen significantly inhibited its levels. The results (Figure 6D) showed that LPS increased the levels of TNF-α (*p* < 0.001), while the doses of 2.5 mg/mL (*p* < 0.01), 5 mg/mL (*p* < 0.001), and 10 mg/mL (*p* < 0.001) of collagen significantly inhibited its levels. The results (Figure 6E) showed that LPS (*p* < 0.001), LPS + collagen 2.5 mg/mL (*p* < 0.001), LPS + collagen 5 mg/mL (*p* < 0.001), and LPS + collagen 10 mg/mL (*p* < 0.001) increased the levels of TGF-β in comparison with the control group. The results (Figure 6F) showed that LPS (*p* < 0.001), LPS + collagen 2.5 mg/mL (*p* < 0.001), LPS + collagen 5 mg/mL (*p* < 0.001), and LPS + collagen 10 mg/mL (*p* < 0.001) increased the levels of VEGF in comparison with the control group.

## 4. Discussion

This is the first study that demonstrated that supplementation with hydrolyzed collagen effectively inhibited the inflammatory response in human skin fibroblasts and keratinocytes under LPS stimulation, as well as induced the expression of pro-collagen-1α, which is a precursor of type I collagen fibers, in a process that involved the synthesis and release of the growth factors TGF-β and VEGF.

It is well known that the transformations that occur in the skin during senescence [3] and in some diseases, such as dermatoses [3], may increase the risk of infections that originate due to a virus [10], bacteria [11], and fungi [12]. In the present study, we demonstrated for the first time that hydrolyzed types I and III collagen importantly inhibited the inflammatory response induced by LPS in human fibroblasts (CCD-1072Sk) and human keratinocytes (hKT-nh-skp-KT0026). Such findings are extremely relevant once the senescence increases the incidence of mostly bacterial infections in the elderly [13]. Therefore, it is plausible to hypothesize that hydrolyzed collagen could be useful for maintaining the immune integrity of the skin. In addition, increased susceptibility to infection was attributed to a process of immunesenescence and inflammaging [13]. Typical examples include Gram-negative bacterial colonization of the oropharynx due to a reduced production of adherence proteins for Gram-positive bacteria and the prevalence of skin colonization by *Proteus mirabilis* and *Pseudomonas aeruginosa* in the elderly, which is increased by approximately 25% compared with adults [13]. So far, although several questions regarding the possible anti-inflammatory effects and the possible mechanisms of action of collagen supplementation remain to be elucidated, a study from Orhan et al. (2021) demonstrated that undenatured type II collagen ameliorated inflammation in a rat model of osteoarthritis [14].

It was reported that cytokines play a key role in the initiation, severity, and duration of the inflammatory process of the skin [15,16]. In addition, the central involvement of fibroblasts [15] and keratinocytes [16] in this process was demonstrated. Therefore, the present study showed for the first time that hydrolyzed collagen inhibited the LPS-induced release of an important and classical panel of pro-inflammatory cytokines (IL-1β, IL-6, IL-8, TNF-α) from human skin fibroblasts (CCD-1072Sk) and human keratinocytes (hKT-nh-skp-KT0026). Thus, here we discuss the role of each of these cytokines in the modulation of skin inflammation and healing. In fact, IL-1β was reported as a potent pro-inflammatory cytokine that is synthesized and released by keratinocytes upon activation of *Staphylococcus aureus* [15] and by fibroblast upon activation of *Cutibacterium acnes* [17], and its increased levels are related to cell death and damage. The present study showed for the first time that hydrolyzed collagen reduced IL-1β secretion by skin fibroblasts and keratinocytes. Similar to IL-1β, IL-6, which is another potent pro-inflammatory cytokine, plays an important role in the initiation and chronification of the inflammatory response of the skin, as well as in wound healing [18]. On the one hand, IL-6 is essential for inducing the initial immune response that aims to eliminate a pathogen; however, an excess of IL-6 may induce a variety of harmful effects, requiring its inhibition to physiological levels to resolve the inflammatory process and the tissue healing properly. Accordingly, the present study showed that hydrolyzed collagen was capable of reducing LPS-induced IL-6 release by skin fibroblasts and keratinocytes. In addition, IL-8, which is a pro-inflammatory cytokine with strong chemotactic activity for neutrophils, is involved mostly in skin bacterial infections, contributing to the exacerbation of inflammation and skin structural cell death [19]. Therefore, in the present study, hydrolyzed collagen reduced LPS-induced IL-8 release, reinforcing its anti-inflammatory properties. TNF-α belongs to a family of cytokines that can kill tumoral cells and possesses very high pro-inflammatory properties [19,20]. In addition, TNF-α was described as a pivotal cytokine that is involved in the pathophysiology of psoriasis and also as a mediator of focal infection in skin lesions [19,20]. Thus, the relevance of hydrolyzed collagen in reducing LPS-induced excessive TNF-α release is guaranteed.

An excessive release of these pro-inflammatory cytokines may activate another important signaling pathway that is involved in inflammation and tissue repair and also in fibrosis in the case of excessive activation, which is modulated by the growth factors TGF-β [20] and VEGF [21]. TGF-β is a classical pro-fibrotic factor that provides a key role in wound healing when present at physiological levels [22]. On the other hand, excessive levels of TGF-β result in an impaired wound-healing process characterized by the dysregulated aggregation of extracellular matrix components, triggering fibrotic scar formation [22]. In the present study, it was found that LPS stimulation plus hydrolyzed collagen in fibroblasts and keratinocytes induced an increase in the expression of TGF-β, demonstrating for the first time that hydrolyzed collagen-induced TGF-β expression. Thus, it is plausible to postulate that hydrolyzed collagen may support skin collagen synthesis, and with TGF-β in skin fibroblasts and keratinocytes, hydrolyzed collagen also induced the expression of pro-collagen-1α in skin fibroblasts. However, as a limitation of the present study, an experiment aiming to prove the possible causal relationship between the hydrolyzed collagen-induced TGF-β and pro-collagen-1α was not performed.

In addition, VEGF is involved in multiple components of wound healing, including angiogenesis and, more recently, epithelialization and collagen deposition [23,24]. Furthermore, VEGF was implicated as a pivotal factor in regulating angiogenesis and inflammation under both physiological and pathological conditions [23,24]. It was demonstrated that VEGF is essential for optimal wound healing [23,24], which depends on fibrovascular tissue formation containing fibroblasts, the synthesis and deposition of collagen, and the formation of new blood vessels, which are hallmarks of an established healing response [23,24]. Here, it was demonstrated for the first time that hydrolyzed collagen in LPS-stimulated cells resulted in increased VEGF expression in both skin fibroblasts and keratinocytes. However, whether such effects may result in improved vascularization in the wound-healing process needs to be further investigated using an in vivo model. Furthermore, a synergistic effect among the growth factors, such as TGF-β and VEGF, in accelerating the healing process was demonstrated [25] and observed in the present study, in which hydrolyzed-collagen-induced a concomitant increase in the expression of TGF-β and VEGF.

## 5. Conclusions

In conclusion, hydrolyzed collagen inhibited LPS-induced inflammation in skin fibroblasts and keratinocytes while improving the synthesis of pro-collagen-1α by skin fibroblasts, as well as inducing the proliferation of skin fibroblasts and keratinocytes.

## Figures and Tables

**Table 1:** Molecular, physicochemical, and microbiological characteristics of the PeptPure^®^ collagen peptides.

Molecular Weight (M. W.) in Daltons (Da)	
Total M. W.	<3 Da
>7000 Da	31.15%
5000 Da–6999 Da	13.99%
4000 Da–4999 Da	46.15%
3000 Da–3999 Da	3.72%
2000 Da–2999 Da	1.58%
<2000 Da	3.41%
**Protein Combustion**	
Protein	100%
Nitrogen combustion	16.48%
Protein factor	6.5
**Aminogram**	
Alanine	9.02%
Arginine	7.53%
Aspartic acid	5.70%
Glutamic acid	10.06%
Glycine	23.61%
Histidine	0.72%
Isoleucine	1.43%
Leucine	2.76%
Phenylalanine	1.91%
Proline	12.90%
Serine	3.29%
Threonine	1.89%
Lysine	3.57%
Tyrosine	0.48%
Valine	2.27%
**Ash in food**	
Ash	1.65 g/100 g
**Cystine and Methionine**	
Cystine	0.03%
Methionine	0.82%
**Heavy metals**	
Arsenic	39.5 ppb
Cadmium	<5.00 ppb
Lead	<5.00 ppb
Mercury	<5.00 ppb
**Hydroxyproline**	
Hydroxyproline	9.49%
**Sodium**	
Sodium (Na)	<66.5 mg/kg
**Pesticide–glyphosate** **compounds**	
Glufosinate	<0.01 mg/kg
Glyphosate	<0.01 mg/kg
**Tryptophan**	
Tryptophan	<0.01%
**Zinc (Zn) in foods**	
Zinc (Zn)	<3.29 mg/kg

## Data Availability

All raw data will be available upon a reasonable request to the corresponding author.
